# Nanoscale Strain Fields Research of Boundaries between B2 Matrix and G.P. Zone in Ni-Ti Alloy Thin Films

**DOI:** 10.1155/2014/127032

**Published:** 2014-03-24

**Authors:** Shilei Zhao, Chunwang Zhao

**Affiliations:** College of Science, Inner Mongolia University of Technology, Hohhot 010051, China

## Abstract

Ti-47at.%Ni alloy films were prepared by magnetron sputtering followed by 460°C for 40 minutes heat-treatment. The strain fields between B2 phase matrix and G.P. zone were mapped by a combination of high-resolution transmission electron microscopy and geometric phase analysis method. It was found that there is a compressive strain region parallel to the longitudinal axis of G.P. zone with 2 nm in width, −2.2% in average strain at the boundaries between B2 phase and G.P. zone.

## 1. Introduction

Shape memory alloy (SMA) with shape memory effect and superelasticity has had an increasing interest for researchers of mechanics of materials in recent decades. With the deepening of theoretical research, the applications of SMA have also made considerable progress including machinery, electronics, chemicals, aerospace, energy, and medical care and many other fields. SMA has a unique solid phase transformation characteristic that is thermoelastic martensitic transformation. The high temperature phase of SMA is austenite, while the low temperature phase of SMA is martensite [1]. The actual SMA is usually in a polycrystalline status, and the grain size and the microstructure are closely related to the thermal treatment history, which often results in a multiphase coexistence in the grain interior of SMA. Thus, there are a lot of grain boundaries and phase boundaries in SMA, which have a critical influence on the mechanical properties of the shape memory material [2].

So far, it has been found that there are AuCd, CuZn, TiNi, and a variety of alloy systems possessing the shape memory effect, wherein the NiTi-based alloys have excellent shape memory effect, biocompatibility, and other characteristics, which have become the most widely used alloy systems. Among Ni-Ti binary alloys are the most representative memory alloy materials. The high temperature austenitic parent phase of Ni-Ti binary alloy is B2 cubic structure, while the low temperature martensite phase is B19′ monoclinic structure [3]. The martensite transformation from high temperature phase to low temperature phase can have two paths. One is 1-stage B2-B19′ transformation, and the other is 2-stage B2-R-B19′ transformation. The occurrence of 1-stage transformation or 2-stage transformation depends on the composition and thermodynamic processing history of Ni-Ti binary alloy. Ni-rich Ni-Ti binary alloy usually forms Ni_4_Ti_3_ precipitates after aging treatment, which has an important impact on the phase transformation. Kim et al. studied the phase transformation before and after heat-treatment in a Ti-50.9at.%Ni alloy. It was found that Ni-rich alloy only occurred 1-stage transformation after solution treatment, while the alloy occurred multi-stages transformation after aging treatment at 473 K. The microstructural analysis showed that the Ni_4_Ti_3_ precipitates appeared during aging treatment, which plays a key role in the phase transformation process. It is because the lattice mismatch strain exists at the boundaries between B2 matrix and Ni_4_Ti_3_ precipitates which results in the R-phase transformation occurrence prior to the martensitic transformation [4]. But for the Ti-rich Ni-Ti binary alloy, the heat-treatment can form two kinds of different precipitates, which are plate-like Guinier-Preston (G.P.) zones and granular Ti_2_Ni. Although they do not affect obviously phase transformation of Ni-Ti binary alloy, the presence of lattice mismatch strain at the boundaries between these precipitates and the matrix will affect the overall mechanical properties of the Ni-Ti binary alloy [5]. So, measurement of the lattice mismatch strain at the boundaries between these precipitates and the matrix is very important. However, the strain is usually only in the region of boundaries with several nanometers width, which is very difficult to measure and analyze. So far, there is still no report in the literature about the strain field measurement of boundaries between matrix and G.P. zone in Ni-Ti binary alloy.

With the development of electron microscopy, transmission electron microscope (TEM) has been becoming a powerful tool for observing the microstructure and measuring the microscopic deformation fields [6]. A number of high-precision transmission electron microscopy-based experimental mechanics methods have been proposed, such as the geometric phase analysis [7], which has been applied to dislocations [8], nanoparticles [9], and other strain field research. It provides an effective measurement method for the strain field of grain boundaries or phase boundaries in Ni-Ti alloy. In this paper, the microstructure of boundaries between matrix and G.P. zone in Ti-rich Ni-Ti alloy was observed by using transmission electron microscopy. The high-resolution transmission electron microscopy (HRTEM) image was taken for the boundaries followed by strain fields mapping of the boundaries using the geometric phase analysis method.

## 2. Materials and Methods

### 2.1. Preparation of Ni-Ti Alloy Thin Films and Heat-Treatment

Ni-Ti binary alloy films were prepared by the vacuum magnetron sputtering, using two elemental targets of Ti-50 at.%Ni and Ti. The substrate was silicon wafer coated with about 100 nm thick silicon nitride film. The base vacuum is 4.7 × 10^−5^ Pa. The thickness of as-deposited films is about 1.3 *μ*m. The composition was determined to be Ti-47at.%Ni by an energy dispersive spectrometry equipped in a scanning electron microscope. Because the as-deposited films were amorphous, the films were carried out a crystallization heat treatment. The substrate with the films was put into a vacuum tube furnace and then heated at 460°C for 40 minutes followed by cooling to room temperature with a fan.

### 2.2. Transmission Electron Microscopy

The films were peeled off the substrate and glued on a 3 mm diameter TEM copper ring with an epoxy. Ar ion milling was applied for the sample at a very small angle until perforation. Then microstructure was observed with a JEM-2010 TEM at 200 kV. The TEM images were recorded with a GATAN 1 k × 1 k CCD and processed by using DigitalMicrograph software. Strain fields were mapped by using the GPA Phase software. The simulation image of electron diffraction of Ni-Ti B2 phase was calculated by using the JEMS software.

## 3. Results and Discussion

**Figure 1:**
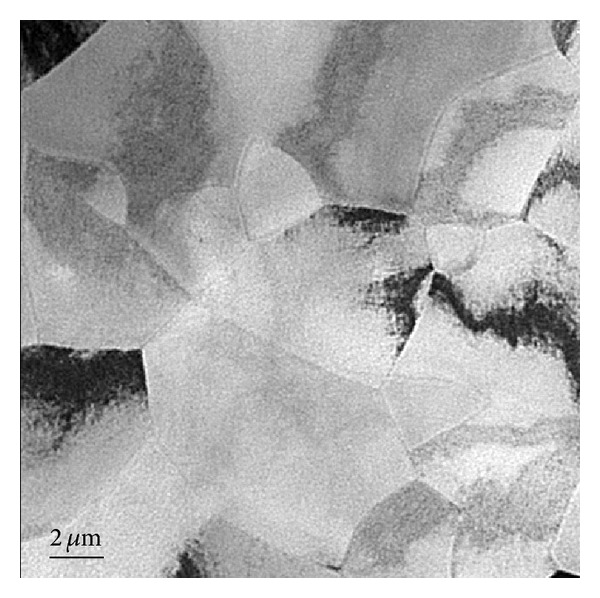
TEM bright field image of Ni-Ti alloy thin films.

Figure 1 depicts the TEM bright field image of Ni-Ti alloy thin films. It can be seen that the Ni-Ti alloy is in polycrystalline status, and the grain size is larger with an average diameter of 3.5 *μ*m. Because of the interaction between grains during growth process, the grain boundaries are in irregular shape and the contrast of the image is not uniform. It is believed that there is a certain distortion or microscopic strain in the grain interior or boundaries.

**Figure 2:**
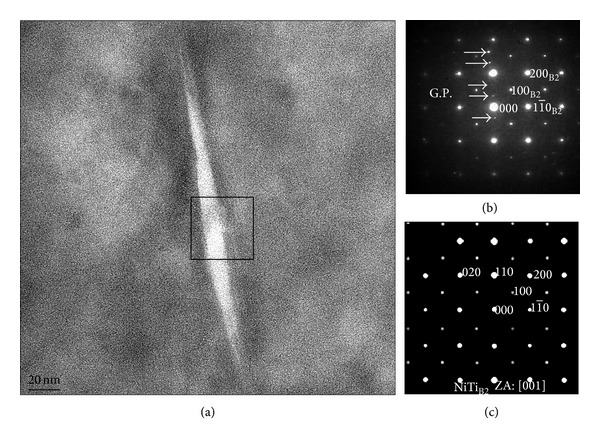
TEM bright field image and electron diffraction image of B2 phase and GP zone in Ni-Ti alloy thin films: (a) TEM bright field image; (b) electron diffraction image of (a); (c) simulated diffraction image of NiTi_B2_ in [001] zone axis.

For analyzing the microstructure of the alloy in more detail, a grain interior region was observed carefully and its TEM bright field image is depicted in Figure 2(a). It is seen that there is a plate-shaped precipitate in the middle region of the image. The length of the precipitate is about 110 nm, while the maximum width is about 9 nm. The electron diffraction pattern of the observed area (Figure 2(a)) is depicted in Figure 2(b). It shows that observed area is approximate in single crystal except those arrowed spots. After comparing with the stimulation image of electron diffraction pattern of NiTi_B2_ in [001] zone axis (Figure 2(c)), the matrix in Figure 2(a) can be determined to be Ni-Ti B2 phase. Those additional diffraction spots marked by arrows do not belong to the NiTi_B2_ diffraction pattern, which indicates they are not from the matrix. Comparing with Figure 1(b) in reference [5], it is found that the positions of these additional diffraction spots are very near in agreement with the so-called G.P. zone diffraction pattern. But in the current work, these diffraction spots deviate <110>_B2_ direction for 5°. This can be explained as follows: since the as-deposited Ti-rich Ni-Ti binary alloy films are amorphous, the heat-treatment is necessary for crystallization. When the atomic fraction of Ti is large, G.P. zones are formed first, and then crystallization occurs. Thus G.P. zone and B2 matrix have no certain orientation relationship [2]. So, the precipitate in Figure 2(a) can be determined to be a G.P. zone, and the matrix is Ni-Ti B2 phase.

**Figure 3:**
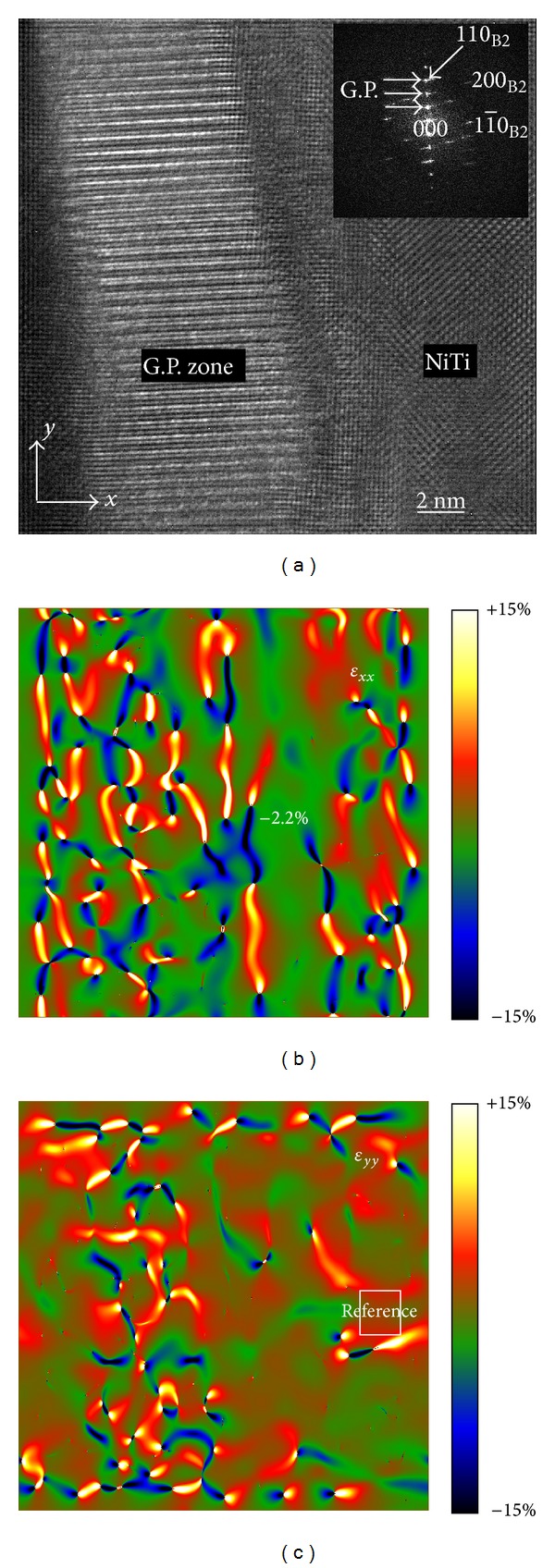
HRTEM image and strain maps of boundaries between B2 phase and GP zone in Ni-Ti alloy thin films (a) HRTEM image; (b) *ε*_*xx*_ strain map; (c) *ε*_*yy*_ strain map.

Figure 3(a) depicts the HRTEM image of boundaries between B2 phase and G.P. zone (boxed area in Figure 2(a)). The inset is the corresponding fast Fourier transformation image of the HRTEM image, which is the same as Figure 2(b). Taking the* x*-axis parallel to ${\left[1\overset{-}{1}0\right]}_{\text{B}2}$ and the* y*-axis parallel to [110]_B2_, then the strain field can be calculated from the HRTEM image (Figure 3(a)). The full-field normal strains *ε*_*xx*_ and *ε*_*yy*_ were mapped, respectively, in Figures 3(b) and 3(c) by using geometric phase analysis method [7]. A reference (boxed area in Figure 3(c)) which is far from the boundaries was selected for the strain field calculation. The different colors in strain field maps represent the different strain values, and the relationship between the colors and the strain values can be identified with the color marker shown in the bottom right area of Figures 3(b) and 3(c). Two diffraction spots of 110_B2_ and ${\left[1\overset{-}{1}0\right]}_{\text{B}2}$ were used for strain field calculation. So, we can only obtain and analyze the strain of boundaries in B2 matrix side. There are a lot of strain concentrated areas in the B2 matrix, which indicate dislocation existence [9]. Meanwhile, strain concentrated areas in the G.P. zone indicate nothing, because only the strains of the B2 matrix that have been calculated.

G.P. zones are Ti-rich regions [2, 5]. The first formation of G.P. zones makes the Ni-rich for surrounding area of G.P. zone, which results in a Ni-rich B2 phase microstructure in a very small area around the G.P. zones during the crystallization process of Ni-Ti alloy films. So, comparing with the normal B2 lattice, the lattice will inevitably shrink in this small area in B2 matrix around the G.P. areas, because Ti atoms are larger than Ni atoms by 19% [2]. As a result, we can get a compressive strain in the small area around the G.P. zone. The experimental measurement result actually has confirmed this deduction. According to Figure 3(b), we can find a small area with a width of 2 nm, average strain of −2.2 % in the B2 matrix besides the boundaries between B2 matrix and G.P. zone. But in Figure 3(c), the strain field is uniform and the strain values are close to 0, which indicates that there is no deformation in* y* direction. As mentioned previously, there is no strictly crystallographic orientation relationship between B2 phase and G.P. zone in current Ni-Ti alloy. But the electron diffraction pattern (Figure 2(b)) and HRTEM image (Figure 3(a)) show that the crystal planes which are normal to the* y* direction in G.P. zone are nearly parallel to the 110_B2_ crystal planes, which results in an approximate semicoherent relationship for B2 phase and G.P. zone in* y* direction. So there is no deformation or strain which can be detected in* y* direction because of the microscopic interactions of these planes. But there is no crystallographic orientation relationship for B2 phase and G.P. zone in* x* direction, which results in a Ni-rich lattice contraction exhibiting compressive strain.

## 4. Conclusions

In this work, Ni-Ti binary alloy films were prepared by magnetron sputtering followed by heat-treatment for crystallization. The microstructure of the alloy films was observed using a transmission electron microscope. It was found that the films were in polycrystalline status with large grains of 3.5 *μ*m in average diameter. Meanwhile, precipitate exists in the grain interior. According to the analysis for electron diffraction pattern and HRTEM image, the matrix was determined to be NiTi_B2_ phase, while the precipitate is G.P. zone. The strain fields of boundaries between B2 phase and G.P. zone were mapped by using geometric phase analysis method. It was found that there is a compressive strain region parallel to the longitudinal axis of G.P. zone with 2nm in width, −2.2% in average strain at the boundaries between B2 phase and G.P. zone.

## References

[1] Otsuka K, Wayman CM (1998). *Shape Memory Materials*.

[2] Otsuka K, Ren X (2005). Physical metallurgy of Ti-Ni-based shape memory alloys. *Progress in Materials Science*.

[3] Huang X, Ackland GJ, Rabe KM (2003). Crystal structures and shape-memory behaviour of NiTi. *Nature Materials*.

[4] Kim JI, Liu Y, Miyazaki S (2004). Ageing-induced two-stage R-phase transformation in Ti-50.9at.%Ni. *Acta Materialia*.

[5] Kajiwara S (1996). Strengthening of Ti-Ni shape-memory films by coherent subnanometric plate precipitates. *Philosophical Magazine Letters*.

[6] Kret S, Ruterana P, Rosenauer A, Gerthsen D (2001). Extracting quantitative information from high-resolution electron microscopy. *Physica Status Solidi B*.

[7] Hÿtch MJ, Putaux JL, Pénisson JM (2003). Measurement of the displacement field of dislocations to 0. 03Å by electron microscopy. *Nature*.

[8] Zhao CW, Xing YM, Zhou CE, Bai PC (2008). Experimental examination of displacement and strain fields in an edge dislocation core. *Acta Materialia*.

[9] Johnson CL, Snoeck E, Ezcurdia M (2008). Effects of elastic anisotropy on strain distributions in decahedral gold nanoparticles. *Nature Materials*.

